# Isolation of Tricin as a Xanthine Oxidase Inhibitor from Sweet White Clover (*Melilotus albus*) and Its Distribution in Selected Gramineae Species

**DOI:** 10.3390/molecules23102719

**Published:** 2018-10-22

**Authors:** Xiao-Xiao Liu, Shi-Wei Sun, Wen-Jing Yuan, Hua Gao, Yue-Yue Si, Kun Liu, Shuang Zhang, Yang Liu, Wei Wang

**Affiliations:** 1 Department of Natural Medicine and Pharmacognosy, School of Pharmacy, Qingdao University, Qingdao 266021, China; lxxlxx19910924@163.com (X.-X.L.); sunsw@qdu.edu.cn (S.-W.S.); yuanwenjingqdu@163.com (W.-J.Y.); gaohuaqy@126.com (H.G.); qdeduzhangshuang@163.com (S.Z.); buckuper@163.com (Y.L.); 2 Department of Drug Metabolism and Analysis, School of Pharmacy, Qingdao University, Qingdao 266021, China; syy2016qd@163.com (Y.-Y.S.); kunliu62@126.com (K.L.)

**Keywords:** tricin, xanthine oxidase, *Melilotus albus* Medic. ex Desr, Gramineae species

## Abstract

Xanthine oxidase, an enzyme present in significant levels in the intestine and liver, metabolizes hypoxanthine to xanthine and xanthine to uric acid in the purine catabolic pathway. An inhibitory compound acting against xanthine oxidase was isolated from sweet white clover (*Melilotus albus*) by bioassay and high-performance liquid chromatography guided separation. It was identified as tricin by spectroscopic analysis. Tricin possessed a potent xanthine oxidase inhibitory activity with an IC_50_ value of 4.13 μM. Further inhibition kinetics data indicated it to be a mixed-type inhibitor and *K*_i_ and *K*_I_ values were determined to be 0.47 μM and 4.41 μM. To find a rich source of tricin, the distribution of tricin in seven different tissues from four Gramineae species was investigated by high-performance liquid chromatography analysis. The highest amount (1925.05 mg/kg dry materials) was found in the straw of wheat, which is considered as a potentially valuable source of natural tricin.

## 1. Introduction

Natural products are being widely accepted as alternative clinical therapies worldwide, compared with currently available therapeutic options [1,2,3]. Furthermore, the utilization of natural resources to develop food, medicine, pharmaceuticals, cosmetics, and others applications is of growing interest [4,5]. However, various natural resources that are less widely used in medicine and culinary applications still lack scientific information about their chemical constituents and biological properties. Therefore, exploring such properties is still an interesting and useful task. *Melilotus albus* Medic. ex Desr, a nitrogen-fixing legume of the family Fabaceae in the genus *Melilotus*, also known as white sweet clover, has been used for both soil improvement and forage production and is considered a valuable honey plant [5,6]. According to the literature data, bioactive compounds and bioactivities of this plant are scanty. As for the chemical constituents, the occurrence of oleanan series-melilotosides I–IV and nonglycosylated soyasapogenol B in the roots was reported. Recent studies have shown that the acetone, ethyl acetate, and ethanol extracts of *M. albus* are lack of antimicrobial, antibiofilm, and antioxidant potentials [5].

The worldwide prevalence of gout ranges from 0.1% to 10% [7]. Gout not only causes severe arthritis pain, it is also associated with premature death, especially from cardiovascular and kidney disease [8]. Overproduction or underexcretion of uric acid lead to elevated serum uric acid levels, termed hyperuricemia, which is the underlying cause of gout. Xanthine oxidase (XO), a complex molybdenum hydroxylase widely distributed in the liver and gastrointestinal tract and consists of two identical subunits 145 kDa, is a key enzyme in purine metabolic pathway, catalyzing oxypurines (hypoxanthine and xanthine) to uric acid [9]. Therefore, XO inhibitors seem to be widely accepted as potential therapeutic agents for treating hyperuricemia [10]. Moreover, XO-reactive oxygen species (ROX) such as superoxide and hydrogen peroxide have been implicated in various chronic diseases [11]. Previously we found the extract of sweet white clover (*M. albus*) to have inhibitory activity on XO. Thus one or more components of sweet white clover might have potential as regulators of uric acid metabolism. In the present study we report the isolation of tricin as an inhibitory compound acting against XO from sweet white clover. However, its concentration in this plant is not sufficient for commercial use. Tricin is mainly found in cereal grains, such as barley, wheat, rice, oat, and maize. Although tricin is dominantly present in free aglycone, the presence of its conjugated forms cannot be neglected since they contribute to the total amount of tricin [12]. This prompted us to investigate the levels of tricin after acid hydrolysis in different tissues of selected Gramineae species.

## 2. Results and Discussion

### 2.1. Bioassay and High Performance Liquid Chromatography Guided Isolation of Tricin

**Figure 1:**
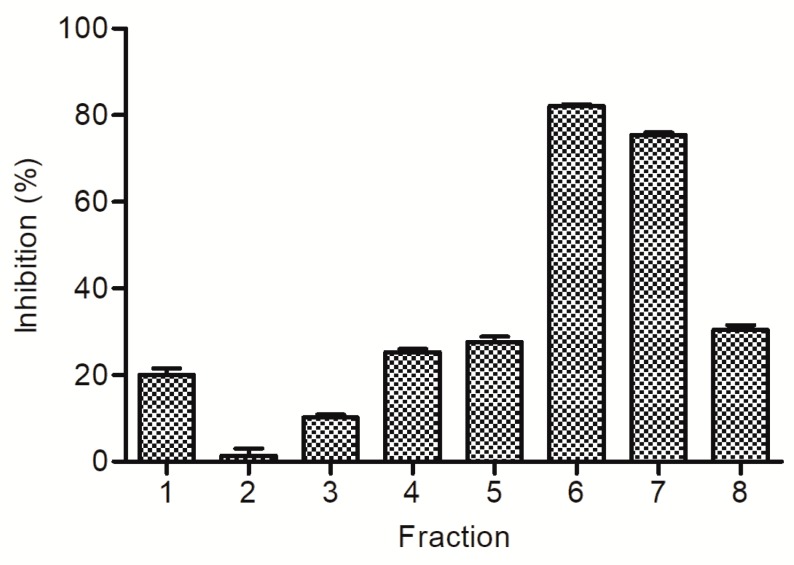
Xanthine oxidase inhibitory activities of the fractions (50 ug/mL) separated from the ethyl acetate-soluble part by RP-18 reversed-phase silica gel open column chromatography. Data represent mean ± S.D. of triplicated experiments.

**Figure 2:**
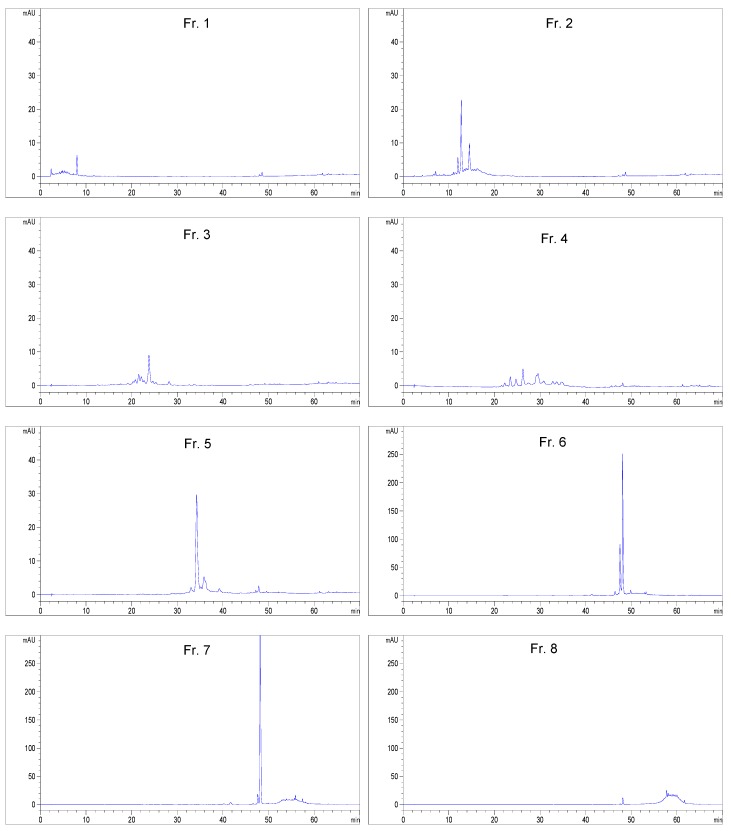
Analytic HPLC chromatograms of fractions **1**–**8** separated from the ethyl acetate-soluble part by RP-18 reversed-phase silica gel open column chromatography.

The inhibitory compound on XO in the whole plant of sweet white clover was isolated with bioassay as an index. The crude extract was separated into four soluble parts by liquid–liquid partition based on the polarities of the components. The soluble parts were tested in the screening assay of XO inhibition, and the petroleum ether-soluble part, ethyl acetate-soluble part, and *n*-butanol-soluble part showed XO inhibitory activity in a dose-dependent manner (Table 1). The water soluble part showed no obvious XO inhibitory activity in all the dose tests. The results showed that the inhibitory activity of petroleum ether-soluble part was almost same as that of *n*-butanol-soluble part, of which the potency was lower than that of crude extract. We also found that the potency of ethyl acetate-soluble part was higher than that of crude extract. This suggests that the inhibitory activity of crude extract was directly correlated to the components in the ethyl acetate soluble part. The ethyl acetate-soluble part was further separated by RP-18 reversed-phase silica gel open column chromatography to afford eight fractions According to the experimental results of bioassay the activities of the fractions are remarkably different (Figure 1). Fractions **6** and **7** showed higher inhibitory activities than those of other fractions. The analytical HPLC chromatograms of each fraction revealed less similarity with each other (Figure 2). On the contrary, the same retention time and different magnitude of main peaks in analytical HPLC chromatograms of fractions **6** and **7** were observed. These fractions were combined and isolated by semipreparative high-performance liquid chromatography. As a result, a compound showing 65.01% inhibition at 20 μM was obtained.

The inhibitory compound was obtained as a light yellow needlelike crystal with MP 289–290 °C. The electrospray ionization-mass spectroscopy (ESI-MS) spectrum showed a positive molecular ion peak at *m*/*z* 331 [M + H]^+^, corresponding to a molecular of C_17_H_14_O_7_, which was further supported by the NMR spectral data. Chemical shift values of the NMR spectra were as follows. ^1^H-NMR (500 MHz, DMSO-*d*_6_, *δ*) 7.31 (2H, s, H-2′, 6′), 6.96 (1H, s, H-3), 6.55 (1H, d, *J* = 1.9 Hz, H-8), 6.20 (1H, d, *J* = 1.9 Hz, H-6), 3.88 (6H, s, H-OCH_3_); ^13^C-NMR (125 MHz, DMSO-*d*_6_, *δ*) 181.7 (C-4), 164.4 (C-7), 163.6 (C-2), 161.4 (C-5), 157.3 (C-9), 148.2 (C-3′, 5′), 139.9 (C-4′), 120.4 (C-1′), 104.4 (C-3), 103.6 (C-2′, 6′), 103.5 (C-10), 98.9 (C-6), 94.2 (C-8), 56.3 (C-OCH_3_). The individual signals in the NMR spectrum of the inhibitory compound were compatible with those of tricin, as reported by Jiao et al. [13].

### 2.2. Distribution of Tricin in Different Tissues of Selected Gramineae Species

**Figure 3:**
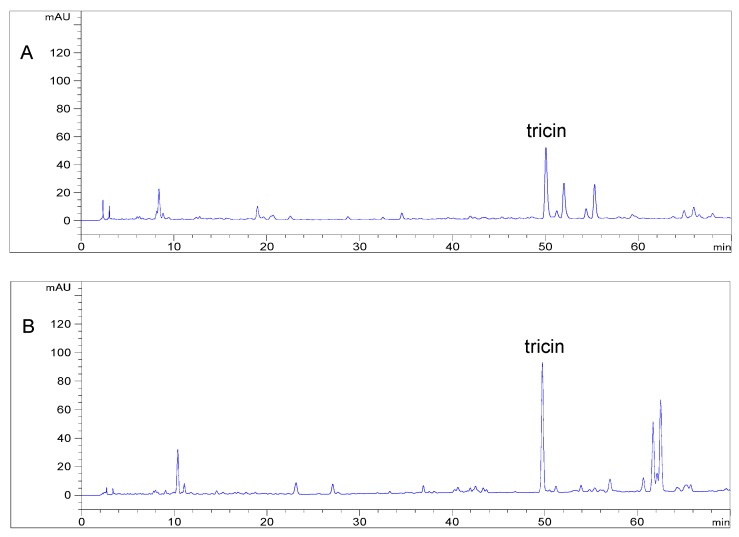
Typical HPLC chromatograms of (**A**) extract from wheat straw before hydrolysis and (**B**) extract from wheat straw after hydrolysis.

Tricin, in the free form, occurs in many monocotyledonous plants such as the family of Gramineae. According to isolation yields reported in the literatures, its content varies from several milligrams to hundreds of milligrams per kilogram of plant material [14]. This prompted us to investigate tricin content in Gramineae species by HPLC method. Seven different tissues from four species were selected for quantitative determination of tricin. Like the free form of tricin, its conjugated forms are also widely distributed in the family of Gramineae [15,16]. Although tricin is dominantly present in free aglycone, the presence of its conjugated forms could not be neglected since they devote to the total amount of tricin. Among the selected tissues from four species, there is only one paper documenting the levels of tricin in wheat hull and bran, in which acid hydrolysis was carried out on extract in order to liberate the free aglycone [12]. The highest content was reported in the bran of medicinal rice Njavara and amounted to 1930.5 mg/kg [17] followed by 772 mg/kg in the hull of wheat Claire and 408 mg/kg in the hull of wheat Bounty [12]. Levels of tricin before hydrolysis and after hydrolysis in different tissues of selected Gramineae species are shown in Table 2. To our surprise, in wheat straw, the concentrations of tricin are significant, ranging from 940.09 mg/kg in dry materials before hydrolysis to 1925.05 mg/kg in dry materials after hydrolysis (Figure 3), which is almost same as that in the bran of medicinal rice Njavara. Furthermore, the content in rice straw with 1143.86 mg/kg dry weight was higher than that in wheat hull. Based on these results and comparisons, wheat straw is considered as a potentially valuable source of natural tricin.

### 2.3. Effect of Tricin on XO Activity

**Figure 4:**
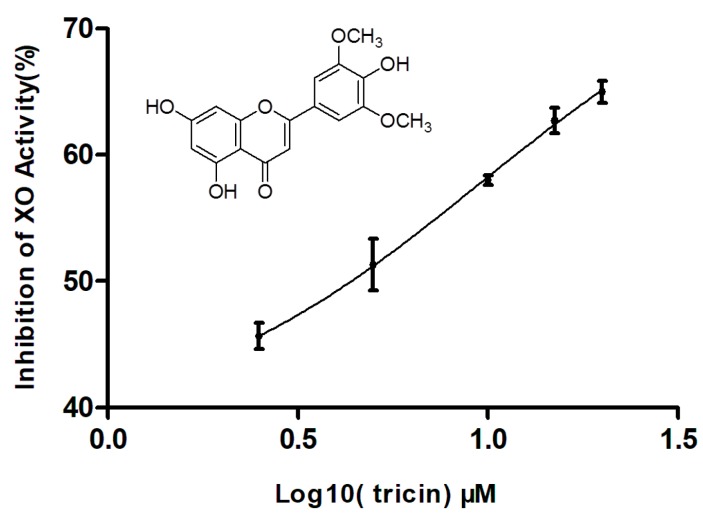
XO inhibitory activity of tricin. Data represent mean ± S.D. of triplicate experiments. Structure of tricin was shown in the inset.

Tricin is a flavonoid-type compound that has diverse properties, including antioxidant, anti-obesity, antidiabetic, anticancer, anti-influenza virus, anti-human cytomegalovirus, anti-inflammatory, antiangiogenic, antifungal, anti-adipogenic, insecticidal, and allelopathic activities [14,18,19,20]. However, it has not been reported to have inhibitory activity on XO. As shown in Figure 4, tricin exhibited xanthine oxidase inhibitory activity with an IC_50_ value of 4.13 μM, comparable to that 2.07 μM of the known XO inhibitor allopurinol, used as a reference standard. So far, many natural flavonoid compounds have been reported to have XO inhibitory activity in vitro [21,22,23,24]. According to Nguyen et al., flavonoid compounds having a methoxy substituent either at C-3′, C-4′, or 5′ in ring C displayed two- to ten-fold stronger activity than without a methoxy group [25]. The present of a methoxy at C-4′ in the ring C of tricin promotes the XO inhibitory activity. XO is also a main biological source of ROS, which cause DNA oxidation, protein degradation, and lipid peroxidation [11]. Thus, the inhibitory activity on XO may have simultaneously antihyperuricemia and antioxidant properties with therapeutic interest. Moreover, compared with nonmethylated flavonoids, methylated flavonoids show relatively high intestinal absorption and metabolic stability [26]. Further in vivo research will be carried out to evaluate the potential of tricin as antigout and antioxidant agent.

### 2.4. Determining the Type of Inhibition by Lineweaver-Burk Analysis

**Figure 5:**
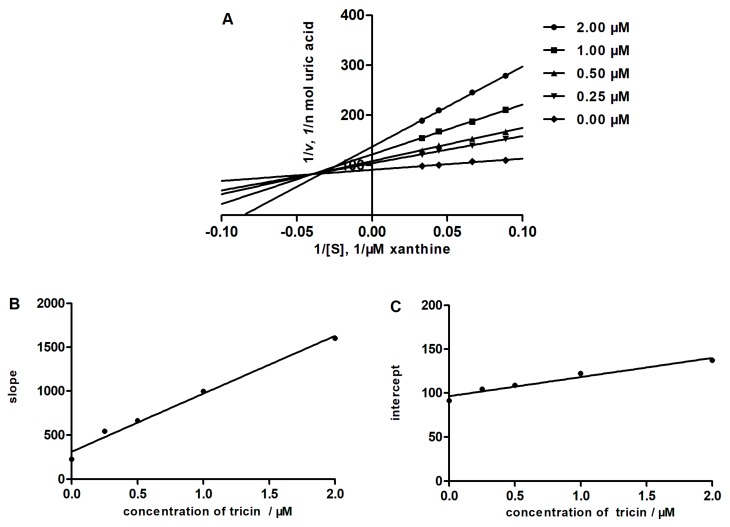
Inhibition kinetics of xanthine oxidase by tricin. (**A**), Lineweaver–Burk plots. The concentrations of tricin are 0, 0.25, 0.50, 1.00, and 2.00 μM. Each point is the average value from triplicate experiments. The secondary plots to calculate the inhibition constants are shown in (**B**) (*K*_i_) and (**C**) (*K*_I_).

In order to obtain the enzyme inhibition mode of tricin, the Lineweaver–Burk plots were established. As shown in Figure 5, the Lineweaver–Burk plots of tricin cross to the left of the 1/v axis but above the 1/[S] axis, which suggested that tricin was a mixed type inhibitor. In mixed inhibition, the inhibitor can not only combine with the free enzyme, but also with the enzyme–substrate complex. The dissociation constant of the enzyme of the inhibitor complex, *K*_i_, can be calculated from the slope of the inhibited curve, and the dissociation constant of the enzyme–substrate–inhibitor complex, *K*_I_, can be calculated from the y-intercept of the inhibited curve. The *K*_i_ and *K*_I_ of tricin were determined to be 0.47 μM and 4.41 μM, respectively. Parallel studies with allopurinol showed that the inhibition mode of allopurinol was of the competitive type and *K*_i_ was 1.92 μM.

### 2.5. Computational Docking Analysis

**Figure 6:**
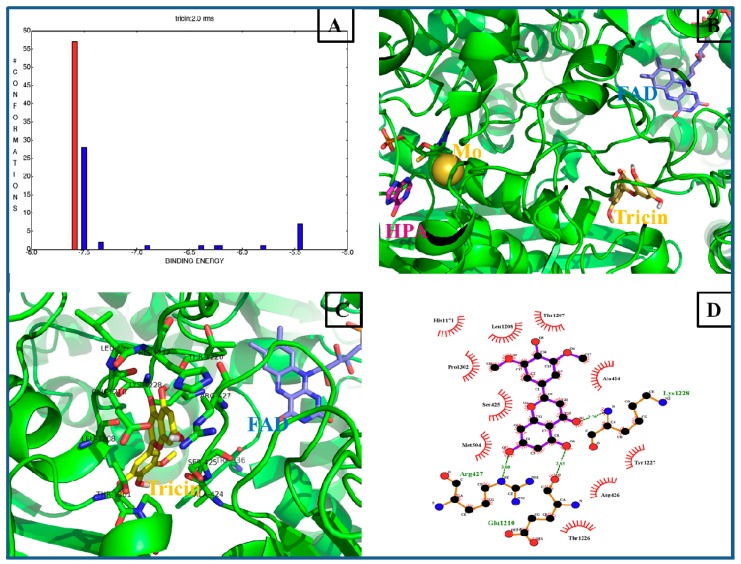
Molecular docking results of tricin to XO. (**A**) Cluster analyses of the AutoDock docking runs of tricin with 3NRZ. (**B**) The binding active positions of hypoxanthine and tricin with 3NRZ. (**C**) Analysis of docked tricin bound to 3NRZ showing the key interactions in the binding pocket. (**D**) Ligplot of tricin bound to 3NRZ showing the key H-bonds (<3 Å) and hydrophobic interactions.

In recent years, a number of molecular docking studies were successful performed to provide insights into the action mechanism of potential small molecules to XO [27,28,29,30]. To provide the possibility of binding interactions between the tricin and XO receptors, molecular docking studies were performed in the present research. The crystal structures of the XO enzyme (PDB ID and 3NRZ) from fresh, unpasteurized bovine milk were used for the docking calculations [31]. For validation of the credible docking method, the natural ligands (hypoxanthine) were docked back into the relevant protein as a control until the root mean square deviation (RMSD) of the best-docked ligand conformation was less than 2.0 Å [11]. The docking results showed that a total of nine conformational clusters were obtained from 100 docking runs at a RMSD tolerance of 2.0 Å, and the highest number (57 out of 100) cluster was found to have the lowest binding energy (−7.59 kcal/mol, red histogram in Figure 6A). In particular, ligand hypoxanthine and potent inhibitor tricin reside at the different receptor pocket of XO (Figure 6B), indicating that the two compounds own the disparate binding interactions. As shown in Figure 6B,C, tricin inserted into the hydrophobic pocket of 3NRZ, which was a long and narrow channel to the flavin adenine dinucleotide (FAD) reaction site, where the reduction of the substrate O_2_ occurs in the presence of XO [27]. The docking results for tricin suggest that three H-bonds and ten hydrophobic interactions with adjacent residues play key roles in successful docking (Figure 6D). The hydroxyl group of tricin at position 7 in a ring was found to form H-bonds with Glu1210 and Arg427 of XO, respectively. The carbonyl group in B ring was connected with Lys1228. In addition, it was found that the C ring of tricin inserted into the hydrophobic region of XO, and interacted with key hydrophobic contacts of residues His1171, Thr1207, Leu1208, Pro1302, and Ala424. These interactions play an important role in the binding of XO and suggest that the inhibitory mechanism of tricin on XO may be due to the compound occupy the active site around isoalloxazine ring in the FAD domain, therefore obstructing diffusion of O_2_^−^ out of the FAD site and causing the formation of H_2_O_2_ [32,33].

## 3. Materials and Methods

### 3.1. General Experimental Procedures

Mass spectra were obtained using a Bruker micro-TOFQ mass spectrometer (Bruker Daltonics, Bremen, Germany). Nuclear magnetic resonance spectra were acquired with a Bruker AV-500 FT-NMR spectrometer operating at 500.1 MHz for ^1^H and at 125.8 MHz for ^13^C at 25 °C; chemical shifts are expressed in *δ* referring to the residual solvent signals *δ*_H_ 2.50 and *δ*_C_ 39.5 for DMSO-*d*_6_, coupling constants, *J.*, are in hertz. All chemical shifts are given in ppm. Column chromatography was performed over a RP-18 reversed-phase silica gel (S-50 μm; YMC, Kyoto, Japan). Analytical HPLC (Agilent technologies, Santa Clara, CA, USA) was performed on an Agilent 1260 system equipped with a G1311C quaternary pump, a G1329B autosampler, a G1316A thermostated column compartment, and a G1314F variable wavelength detector coupled with an analytical workstation. Semipreparative HPLC was performed on an Agilent ProStar SD-1 pump connected with an Agilent ProStar 320 UV–Vis detector (at 360 nm), utilizing a Shim-Pack PREP-ODS column (250 mm × 21.2 mm, i.d., 10 μm, Shimadzu, Kyoto, Japan). HPLC-grade water was purified using a Milli-Q system (Millipore, Boston, MA, USA). All solvents used for the chromatographic separations were distilled before use.

### 3.2. Chemicals

Xanthine, XO, and allopurinol were obtained from Sigma-Aldrich Chemicals (St. Louis, MO, USA). Chromatographic grade methanol and acetonitrile were purchased from Tedia Company (Fairfield, OH, USA). Methanol, ethanol, hydrochloric acid, petroleum ether, ethyl acetate, *n*-butanol, and dimethyl sulfoxide were analytical grade (Beijing Chemical Industry Group, Beijing, China).

### 3.3. Plant Material

The dried whole plant of *Melilotus albus* Medic. ex Desr was purchased from Yonggang Pharmaceutical Company, Bozhou, Anhui province of China, authenticated by Prof. Baomin Feng, School of Life Science and Technology, Dalian University. Rice hull, rice straw, wheat hull, wheat straw, wheat bran, barley bran, and sorghum bran were obtained at the local market, and authenticated by Prof. Yingxia Li, School of Pharmacy, Qingdao University. Voucher specimens (MA-2015-6-0001 and Tricin-S-0001-0008) were deposited at the Department of Natural Medicine and Pharmacognosy, School of Pharmacy, Qingdao University, China.

### 3.4. Extraction and Isolation

After being cut into pieces, the dried (1 kg) whole plant was reflux extracted with 70% ethanol twice (2 h each time). The 70% ethanol extract was concentrated under reduced pressure to obtain a dried extract (180 g). The dried extract (100 g) was suspended in water and successively partitioned with petroleum ether, ethyl acetate, and *n*-butanol to give a petroleum ether-soluble part (6.4 g), ethyl acetate-soluble part (7.0 g), n-butanol-soluble part (6.4 g), and water soluble-part (78.2 g). Based on the results of the XO inhibitory activities of these soluble parts, the ethyl acetate-soluble part (2 g) was subjected on a RP-18 reversed-phase silica gel and eluted with mixtures of CH_3_OH and H_2_O (40:60, 60:40, and 80:20, successively), yielding eight fractions (Fractions **1**–**7**) on the basis of thin-layer chromatography analyses. After analysis of the HPLC chromatograms and XO inhibitory activities of these fractions, Fractions **6** and **7** were combined and isolated by semipreparative high-performance liquid chromatography employing CH_3_OH-H_2_O (48:52) as the mobile phase at a flow rate of 2.4 mL/min to yield tricin (86 mg).

### 3.5. HPLC Analysis of the Fractions

HPLC analysis of the fractions separated from ethyl-acetate soluble part by RP-18 reversed-phase silica gel open column chromatography was performed as follows. The solution of each fraction was prepared by dissolving 20 mg in 10 mL of methanol, and filtered through a 0.45 μm membrane filter (Jinteng Nylon66, Tianjin Jinteng Experimental Equipment, Tianjin, China), and 5 μL of the solution was subjected to HPLC under the following conditions. Samples were separated on a XDB-C18 (250 mm × 4.6 mm, 5 µm, Agilent Technologies, Santa Clara, CA, USA). The chromatographic separation was performed with methanol (A) and water containing 0.1% phosphoric acid (B) as the mobile phase. A gradient program was used: 0–10 min, linear change from A-B (30:70, *v*/*v*) to A-B (40:60, *v*/*v*); 10–40 min, linear change from A-B (40:60, *v*/*v*) to A-B (50:50, *v*/*v*); 40–60 min, linear change from A-B (50:50, *v*/*v*) to A-B (100:0, *v*/*v*); and 60–70 min, isocratic elution with A. The column temperature was maintained at 25 °C, and the flow rate was 1.0 mL/min. The fractions were detected by monitoring UV absorption at 360 nm.

### 3.6. XO Inhibitory Activity Assay and XO Inhibitory Modes of Action Assay

The XO inhibition assays and the inhibitory mode assay of the extract, soluble parts, fractions, and tricin were performed according to the method modified by our group [34,35].

### 3.7. Quantitative Determination of Tricin

Rice hull, rice straw, wheat hull, wheat straw, wheat bran, barley bran, and sorghum bran were ground into powder using an electric disintegrator (FW177, Tianjin Taisite Instruments, Tianjin, China) and passed through a 60 mesh stainless steel sieve for homogenization. The powder was stored in airtight bags at 4 °C for later use. The 10 g sample was accurately weighed and moved into a round-bottomed flask equipped with a water condenser tube. Each sample was reflux extracted with 100 mL 70% aqueous methanol for 6 h at 85 °C. The solvent-free extract was subjected to hydrolysis by methanol–15% hydrochloric acid solution (4:1, *v*/*v*, 25 mL) in a thermostat boiling-water bath at a temperature of 85 °C for 15 min. The hydrolyzed sample was cooled to room temperature. Each solution was filtered with membrane filter, and the solution was subjected to HPLC under the following conditions. The mobile phase was a mixture of acetonitrile (A) and water containing 0.5% phosphoric acid (B). The gradient elution program was 0–15 min, linear change from A-B (15:85, *v*/*v*) to A-B (20:80, *v*/*v*); 15–20 min, isocratic elution with A-B (20:80, *v*/*v*); and 20–70 min, linear change from A-B (20:80, *v*/*v*) to A-B (40:60, *v*/*v*). The flow rate was 1.0 mL/min. A Generall-M C18 (250 mm × 4.6 mm, 5 µm, Guangzhou Yanchuang Biotechnologies, Guangzhou, China) was used at 25 °C. The detection wavelength was monitored at 350 nm. The standard stock solution (977.6 ug/mL) of tricin was prepared in methanol. The calibration curve contained ten concentrations and was established with a range of 9.8 to 977.6 µg/mL. Quantitative determination was performed by the external standard method. The data are presented as the mean value ± standard deviation of three replicated analyses of samples.

### 3.8. Docking Studies

Molecular docking studies between tricin, natural ligand and xanthine oxidase enzyme were performed by a series of software programs: ChemBioOffice 2010 (PerkinElmer, Waltham, MA, USA, version 12.0), Autodock (The Scripps Research Institute, La Jolla, CA, USA, version 4.2.6), LigPlot^+^ (European Bioinformatics Institute, Cambridge, UK, version v1.4), and PyMol (Schrödinger, New York, NY, USA, version 1.5.0.3). The X-ray crystal structure of XO (PDB ID: 3NRZ, resolution 1.8 Å) used as target receptor protein was download from the Protein Data Bank (http://www,rcsb.org/pdb). The natural ligand hypoxanthine (HPA) used as control and the inhibitory compound tricin were minimized with ChemBio3D Ultra (PerkinElmer, Waltham, MA, USA, version 12.0). Docking studies were performed using a modification of a previously reported method [31]. In brief, all the water molecules, co-crystallized ligands, and J, K, and L chains of 3NRZ were eliminated, the hydrogen atoms were added, and the Gasteiger charges were computed. The grid box was set at 100 Å ×1 00 Å × 100 Å with the spacing of 0.375 Å, and the docking parameter files were created using the Lamarckian genetic algorithm (GA) before the program was run. The results of the docking computations were ranked by binding energy. The PyMol molecular graphic system and LigPlot were used to visualize the conformations and interactions between the ligands and the target proteins.

### 3.9. Statistical Analysis

Data from the XO inhibitory assay were expressed as the mean ± standard deviation (S.D.). IC_50_ value, inhibitory type, and *K*_i_ and *K*_I_ values were analyzed using GraphPad Prism 5.0 software (GraphPad Software, La Jolla, CA, USA).

## 4. Conclusions

An inhibitory compound, tricin, acting against xanthine oxidase was isolated from sweet white clover (*M. albus*) by bioassay and high-performance liquid chromatography guided separation. The straw of wheat was found to be a rich source of tricin. The abundance of tricin in wheat straw should facilitate the effective utilization of this compound.

## Figures and Tables

**Table 1:** XO inhibitory effects of the extract and soluble parts.

	XO Inhibition (Mean ± S.D.%)		
	200 μg/mL	100 μg/mL	50 μg/mL
70% ethanol extract	38.26 ± 1.07	21.19 ± 1.14	8.33 ± 0.69
Petroleum ether-soluble part	27.84 ± 1.00	17.12 ± 1.97	12.69 ± 2.15
Ethyl acetate-soluble part	66.93 ± 1.52	57.00 ± 1.65	42.26 ± 0.79
*n*-Butanol-soluble part	18.11 ± 1.41	10.23 ± 0.29	N.I. ^1^
Water-soluble part	N.I.	N.I.	N.I.

^1^ N.I., no inhibition.

**Table 2:** Tricin content in different tissues of selected Gramineae species.

Species	Plant Parts	Common Name	mg Tricin/kg Dry Weight (Mean ± S.D.%)	
			Before Hydrolysis	After Hydrolysis
*Oryza sativa*	hull	rice hull	155.16 ± 1.03	188.32 ± 2.27
	straw	rice straw	722.78 ± 22.82	1143.86 ± 54.70
*Triticum aestivum*	hull	wheat hull	511.35 ± 15.80	869.98 ± 33.76
	straw	wheat straw	940.09 ± 13.50	1925.05 ± 17.89
	bran	wheat bran	N.D. ^1^	N.D.
*Hordeum vulgare*	bran	barley bran	33.14 ± 2.44	36.82 ± 0.28
*Sorghum bicolor*	bran	sorghum bran	N.D.	N.D.

^1^ N.D., not detected.
